# Minimally Invasive and Fast Diagnosis of Gastric Cancer Based on Maspin Levels in Different Biological Samples

**DOI:** 10.3390/diagnostics13111857

**Published:** 2023-05-26

**Authors:** Alexandru Adrian Bratei, Raluca-Ioana Stefan-van Staden

**Affiliations:** 1 Faculty of Chemical Engineering and Biotechnologies, University Politehnica of Bucharest, 011061 Bucharest, Romania; brateialexandru@yahoo.com; 2 Laboratory of Electrochemistry and PATLAB, National Institute of Research for Electrochemistry and Condensed Matter, 060021 Bucharest, Romania

**Keywords:** gastric cancer, maspin, stochastic microsensor, biomedical electroanalysis

## Abstract

(1) Background: Human SERPINB5, commonly known as maspin, has diverse functions as a tumor suppressor. Maspin has a novel role in cell cycle control, and common variants were discovered to be associated with gastric cancer (GC). Maspin was proven to also affect the EMT and angiogenesis of gastric cancer cells via the ITGB1/FAK pathway. Information about the maspin concentrations correlated with different pathological features of the patients may facilitate the fast diagnosis and personalized treatment of patients. The novelty of this study is given by the correlations established for the maspin levels in different biological features and clinicopathological features. These correlations can be extremely useful for surgeons and oncologists. (2) Patients and methods: Patients with clinical and pathological features, given the small number of samples available for this study, were selected from the database of the project GRAPHSENSGASTROINTES, and used in accordance with the Ethics Committee approval nr. 32,647/2018 awarded by the County Emergency Hospital from Targu-Mures. Stochastic microsensors were used as new screening tools for the determination of the concentration of maspin in four types of samples: tumoral tissues, blood, saliva and urine. (3) Results: The results obtained using the stochastic sensors were correlated with those tabulated in the clinical and pathological database. A series of assumptions regarding the values and practice important features for surgeons and pathologists were made. (4) Conclusions: This study provided a few assumptions regarding the correlations between the values of maspin levels in the analyzed samples and the clinical and pathological features. These results may be useful as preoperative investigations in order to help surgeons localize, approximate and choose the best treatment. These correlations may facilitate minim invasive and fast diagnosis of gastric cancer based on reliable detection of maspin concentration in biological samples (tumoral tissues, blood, saliva and urine).

## 1. Introduction

Gastric cancer (GC) is the second cause of cancer death after the lung cancer [1] and its tumorigenesis and progression represent a multistage process related to a multifactorial etiology which mainly results from gene-environment interactions. Son et al. [1,2] demonstrated that the expression of maspin (a member of serpin family, known for its tumor suppressor activity) in gastric carcinoma is upregulated. Wang and Chang [3] demonstrated that maspin suppresses cell invasion and migration in gastric cancer through inhibiting EMT (epithelial–mesenchymal transition) and angiogenesis via the ITGB1/FAK pathway; results were based on the immunohistochemical determination of maspin correlated with the expression of ITGB1, FAK, E-cadherin, vimentin, D2-40, and CD34. Maspin, genetically and functionally, was associated with gastric cancer by regulating cell cycle progression [4]. Wang et al. [1,5] found that down-regulated maspin expression is a late molecular event in gastric carcinogenesis; reduced expression of maspin contributes to the progression of gastric cancer. Terashima et al. shown that gene expression profiles in human gastric cancer, e.g., the expression of maspin, correlates with lymph node metastasis [1,6]. Deng et al. [1,7] demonstrated that the down-regulated expression of maspin and the up-regulated expression of uPA and MMP-7 play important roles in the invasion and metastasis of gastric carcinoma; accordingly, they can serve as biomarkers for the biopathological behavior of gastric tumors. Overall, maspin has proved to have a significative role in cancer diagnosis and prognosis [1,8,9,10,11,12]. The expression of maspin was also used for the determination of the efficiency of treatment of gastric cancer, e.g., using fluorouracil-based chemotherapy [13]. All of these studies have mainly taken into account the results of immunohistochemical determinations of maspin, which are semiquantitative determinations of maspin based on colorimetric methods.

The novelty of this study is given by the series of correlations which have been established between maspin levels and clinicopathological features, which can become an extremely useful investigation both for surgeons and for oncologists. The reliable quantification of maspin, and correlation of its concentration with different pathological features of patients, may bring about a simple screening test of whole blood, urine or saliva to quickly diagnose0 gastric cancer. Therefore, this paper proposed the quantification of maspin using stochastic microsensors [14,15], able to perform both the identification and reliable quantification of maspin in different biological samples (tumor tissue, whole blood, urine, saliva) from patients confirmed with gastric cancer, and the correlations of the values of concentrations with different pathological features extracted from the database of the GRAPHSENSGASTROINTES project. The selection of stochastic microsensors as tools and of a stochastic method as a screening method for the identification and quantification of maspin was based on their early utilization for biomedical analysis of different biomarkers and as tools in the screening of these biological samples [16,17,18]. 

## 2. Materials and Methods

### 2.1. Patient Description

Samples were collected from 39 patients as follows: 30 whole blood samples, 32 tumor tissue samples, 29 urine samples and 28 saliva samples. All samples were selected from the database of the GRAPHSENSGASTROINTES project. All biological samples (whole blood, tissue samples, urine and saliva) were collected according to the protocols set out in the Ethics Committee approval number 32647/2018, awarded by the County Emergency Hospital from Targu Mures. Informed consent was provided by all of the patients. Due to the fact that the number of patients was not large, bigger studies to confirm the assumptions made in this article will need to be performed.

### 2.2. Reagents

All chemicals were of analytical grade. The maspin was purchased from Sigma Aldrich and the paraffin oil ($d_{4}^{20}$, 0.86 g/cm^3^) was purchased from Fluka. The design and calibration of the stochastic microsensors used for the determination of maspin’s concentrations in different biological samples were described previously [14]: NS co-doped graphene powder was mixed with paraffin oil until a homogeneous paste was obtained. This paste was mixed with a solution of α-cyclodextrin (1 × 10^−3^ mol L^−1^) at a ratio of 1:1 (mg:μL) to obtain the modified paste. Silver wire served as a contact between the modified paste and the external circuit. The modified paste was placed in non-conducting plastic tubes with an inner diameter of 150 μm and a length of 5 mm. The stochastic microsensors were washed with deionized water and dried between measurements. When not in use, they were kept in a dry place.

### 2.3. Apparatus

For all determinations of the concentrations of maspin, an Autolab PGSTAT 302 (from Metrohm, Utrecht, The Netherlands) which was connected to a computer equipped with GPES software was used [14]. The electrochemical cell included the reference electrode (Ag/AgCl), the stochastic microsensor and the auxiliary electrode (Pt) [14].

### 2.4. Methodology

**Scheme 1:**
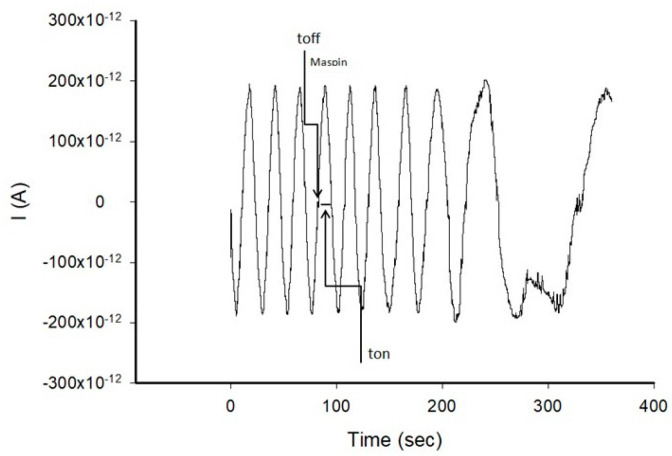
An example of the assay of maspin in biological samples using the stochastic microsensor.

The stochastic method based on the utilization of the chronoamperometric technique was used for all determinations of maspin. All measurements were carried out at 25 °C. The samples were used as obtained from the patients; no processing was necessary before the measurements. The stochastic microsensor together with the Ag/AgCl wire and the Pt wire were introduced into the sample. The values of t_on_ and t_off_ (signature of maspin) were determined at a constant potential (125 mV vs. Ag/AgCl). Based on the signature of maspin (value of t_off_), maspin was identified in the diagrams recorded with the stochastic microsensors (the signature value was 2.8 s), and the value of t_on_ was read in the diagrams (Scheme 1) in between two t_off_ values and used for the determination of concentration of maspin according to the equation of calibration obtained at two values of pH: 3.00 (for urine analysis), and 7.40 (for whole blood, tumor tissue, and saliva analysis) [14]:

1/t_on_ = 0.03 + 3.82 × 10^4^ × C for pH = 7.40      <C> = g mL^−1^, < t_on_> = s

1/t_on_ = 0.04 + 2.96 × 10^3^ × C for pH = 3.00      <C> = g mL^−1^, < t_on_> = s

At pH = 7.40, the working concentration range was between 4.1 fg mL^−1^ and 2 µg mL^−1^, and the RSD (reproducibility of the measurements; the lower the value, the better the precision) for measurements recorded in biological samples was lower than 1.00%. The limit of quantification was 4.1 fg mL^−1^.

At pH = 3.00, the working concentration range was between 20.5 pg mL^−1^ and 2 µg mL^−1^, and the RSD (reproducibility of the measurements; the lowest the value, the better the precision) for measurements recorded in biological samples was lower than 1.00%. The limit of quantification was 20.5 pg mL^−1^.

## 3. Results

The concentrations of maspin in different biological samples obtained using the stochastic method were correlated with a series of pathological features as described below (Table S1).

Correlation of the concentration of maspin in biological samples with the location of the tumor: Ten of the patients were diagnosed with proximal gastric cancer, and it was observed that these patients are linked to higher values of maspin in urine and lower values in saliva. For proximal location, the mean values recorded for maspin concentrations were: 122.48 pg/mL in whole blood samples, 48.83 pg/mL in tumoral tissue samples, 627.88 pg/mL in urine samples, and 30.72 pg/mL in saliva samples. For a better view, the patients were classified according to the concentration of maspin (Table 1).

As can be observed, in urine samples, most maspin concentrations (5 of 8) were high (>150 pg/mL) compared to the other 3 sample types, while in saliva samples, most maspin concentrations (6 of 8) were low (<30 pg/mL).

For middle gastric cancer location, the mean values recorded for the concentration of maspin were: 100.73 pg/mL in whole blood, 104.89 pg/mL in tumoral tissue, 381.64 pg/mL in urine, and 66.61 pg/mL in urine. For a better view, the patients were counted according to maspin concentration (Table 2).

For a distal location of gastric cancer, the mean values recorded for maspin were: 40.92 pg/mL in whole blood, 44.00 pg/mL in tumoral tissues, 365.70 pg/mL in urine, and 44.89 pg/mL in saliva. For a better view, the patients were counted as a function of concentration of maspin presented by patients presenting a distal location (Table 3). As it can be observed for a distal location, in tissue samples, most of the maspin concentrations (13 of 16) were low (<30 pg/mL); accordingly, this location can be linked to low values in tissue samples.

Tissular maspin concentrations are lower for proximal and distal locations, and tissular maspin concentrations lower than 30 pg/mL are linked to distal gastric cancer; for whole blood samples, there is a tendency of the maspin concentrations to decrease from proximal to distal. The urinary maspin’s decrease tendency is maintained and the highest urinary maspin concentrations (>150 pg/mL) are linked to distal or proximal locations, while for the salivary maspin concentrations, the values recorded are the lowest and salivary maspin concentrations lower than 30 pg/mL are linked to distal or proximal locations. One can conclude that determining the tissular maspin concentration and salivary and urinary maspin concentrations can reliably indicate the location of gastric cancer.

Correlation of the maspin concentrations with the maximum diameters of the tumors: The maximum diameter of tumors vs. maspin concentrations determined in different types of biological samples is given in Table S1. As can be observed in Figure S1, the maspin concentrations in different biological fluids are only slightly correlated, and therefore we analyzed a linear combination of tissular maspin, whole-blood maspin, urinary maspin, and salivary maspin to find a parameter (marked as S parameter) able to correlate with the tumor diameter.

**Figure 1:**
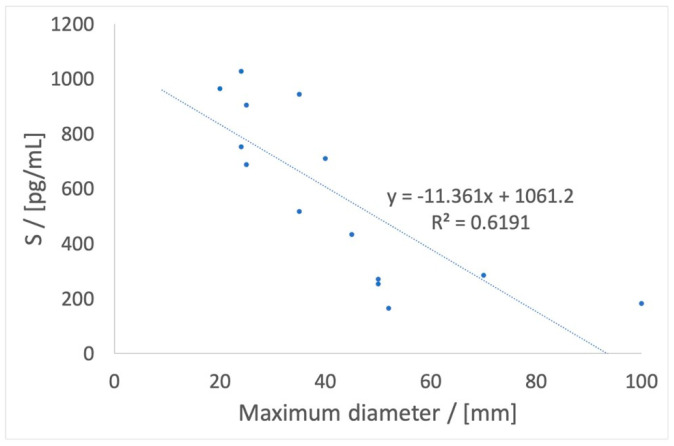
Correlation of S parameter to the maximum tumor diameter.

While the maspin concentration is higher in tumoral tissue, whole blood and urine, a linear combination of the concentrations of tissular maspin, whole-blood maspin, and urinary maspin gave the following equation, named the S parameter equation (Spe):

S (pg/mL) = 2.1 × [Maspin]_whole blood_ + 1.45 × [Maspin]_tissue_ + 1.05 × [Maspin]_urine_

where S (pg/mL) is a parameter calculated using the tissular maspin concentration, whole-blood maspin concentration, and urinary maspin concentration. [Maspin]_whole blood_ is the whole-blood maspin concentration, [Maspin]_tissue_ is the tissular maspin concentration, and [Maspin]_urine_ is the urinary maspin concentration. The S (pg/mL) parameter has a good correlation (*p* = 0.00084) with the tumor diameter, as seen in Figure 1. Accordingly, the S parameter can be used to demonstrate the correlation of the maspin concentrations with the maximum tumor diameter.

Correlation of the maspin concentrations with the microscopic features: A series of microscopic features were correlated with the maspin concentrations (Table S1). The presence of mucus was correlated with the lower values of maspin concentration in all of the samples (Table S2). In saliva, 60.00% of salivary maspin concentrations associated with the presence of mucus are lower than 20 pg/mL, while in the absence of mucus, there are only 23.53% of cases for which salivary maspin is lower than 20 pg/mL. In whole blood samples, 60.00% of whole-blood maspin concentrations are associated with the presence of mucus, their value being lower than 25 pg/mL, while in the absence of mucus, there are only 25.00% of cases for which whole-blood maspin is lower than 25 pg/mL.

Correlations of maspin concentrations were also established with the grading of gastric cancer (another histopathological parameter) (Table S1). Grade 2 tumors were correlated with lower values of tissular maspin concentration (62.5% of the concentration were lower than 20 pg/mL) compared to grade 3 tumors (only 11.1% of the concentrations were lower than 20 pg/mL). By eliminating the highest value of whole-blood maspin concentration from the grade 3 group, the grade 3 tumors were found to be associated with lower maspin concentrations in whole-blood samples (having as a mean maspin concentration 32.56 pg/mL), compared to grade 2 tumors (having the mean maspin concentration 82.16 pg/mL).

*Correlation of maspin concentration with the TNM staging system:* The pT and pN values associated with the maspin concentrations are given in Table S1. Regarding the pT values, it was observed that most maspin concentrations lower than 55 pg/mL are related to pT1 and pT4 (14 of 17 in whole blood samples and 16 of 24 in tissue samples). A more detailed presentation of results for each pT value in whole blood, tumor tissue, urine, and saliva sample and their division by the value of 55 pg/mL are shown in Table 4.

Regarding pN values (Table S1), these values were correlated with whole-blood maspin concentrations. The increase in pN values was correlated with the decrease in the concentration of whole-blood maspin (Figure S2, Table S3). Average concentration values versus pN are shown in Table S2 and Figure S2.

**Figure 2:**
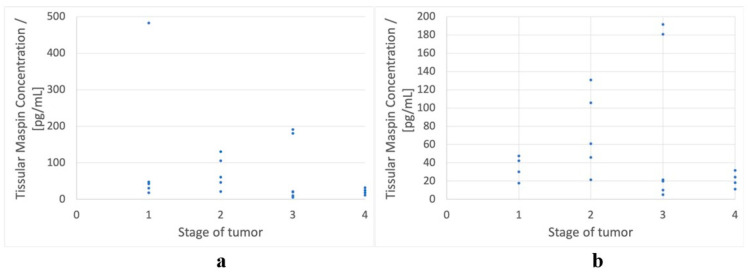
Correlation of tissular maspin concentration with the stage of gastric tumor: **a**. for all values of tissular maspin concentration; **b**. after eliminating the higher value belonging to stage 1 of gastric cancer.

Correlation of the maspin concentrations with the stages of gastric cancer: The association of the maspin concentration with stages of gastric cancer is shown in Table S1. A correlation between tissular maspin concentration and the stage of gastric tumor has been found in the form of a Gaussian-like distribution (Figure 2a). This implies that the central tissular maspin concentrations (corresponding to stages 2 and 3) are higher than the extreme ones (corresponding to stages 1 and 4).

By eliminating the highest value belonging to stage 1 of gastric cancer, a better shape for the Gaussian-like distribution may be seen in Figure 2b. It can easily be observed that excluding one tissular maspin concentration corresponding to stage 1, all other tissular maspin concentrations corresponding to stages 1 and 4 are lower than 60 pg/mL. We observed that there are few tissular maspin concentrations associated with stages 2 and 3 which are lower than 60 pg/mL, but most of the tissular maspin concentrations associated with stages 2 and 3 are higher than 60 pg/mL. Accordingly, we can assume that values higher than 60 pg/mL for tissular maspin concentration exclude stages 1 and 4.

Correlation of the maspin concentration with vascular, lymphatic, and perineural invasions: We analyzed vascular, lymphatic and perineural invasion. All of these were related to the whole-blood maspin concentrations (Table S1). In the case of lymphatic invasion, representing the invasion as a function of whole-blood maspin concentration (Figure S3a), a Gaussian-like distribution was observed. The values of whole-blood maspin concentration correlating with a lack of invasion are more central, and for this we calculated the distance from a reference value for the maspin concentration, considered as 180 pg/mL. To prove that most values are within 80 pg/mL the reference value, a lymphatic maspin equation was proposed (Lme):

Dist = (|180 − [Maspin]_whole blood_|)/80

where Dist represents the distance between the reference whole-blood maspin concentration (180 pg/mL) divided by 80 (which represents the interval within which the concentration of whole-blood maspin can be found), and [Maspin]_whole blood_ is the whole-blood maspin concentration.

It was observed that Dist is lower than 1 for 66.66% of the whole-blood maspin concentrations with no lymphatic invasion and higher than 1 for 88.24% of the whole-blood maspin concentrations when lymphatic invasion was reported (Figure S3b).

In the case of vascular invasion, it was observed that vascular invasion is related to lower whole-blood maspin concentrations (Figure S4). Overall, 81.82% of the patients with invasion have whole-blood maspin concentrations lower than 50pg/mL, while 66.67% of the patients with no invasion have whole-blood maspin concentration higher than 50 pg/mL. A similar assumption can be made for perineural invasion (Figure S5). Overall, 80% of the patients with invasion have values of whole-blood maspin concentration lower than 65pg/mL, while 62.5% of the patients with no invasion have values of whole-blood maspin concentrations higher than 65 pg/mL, with 65 pg/mL being in this case the reference maspin concentration in whole blood.

## 4. Discussion

The location of gastric tumors was related to the concentrations of maspin in whole blood as follows: a concentration of maspin in whole blood lower than 120 pg/mL suggests a distally located tumor, while a concentration of maspin in whole blood higher than 120 pg/mL can exclude it. In the cases of other locations, concentrations of maspin in saliva and urine must be considered, as follows: a concentration of maspin in saliva lower than 30 pg/mL and a concentration of maspin in urine higher than 400 pg/mL indicate a proximal location, while a concentration of maspin in saliva lower than 50 pg/mL and a concentration of maspin in urine lower than 100 pg/mL are related to a middle gastric location. These details lead to an easier method to find the tumor location in order to obtain a biopsy, and the process can be elaborated with further investigations.

The maximum diameter which can lead the surgeon to perform a margin-free resection is easily estimated based on the S-parameter equation, by means of the calculation of the S parameter, which is reliably associated with the maximum tumor diameter. The maspin concentrations also offer information about microscopic features. For example, the presence of mucus was related to whole-blood and tissular maspin concentrations lower than 60 pg/mL; higher values of concentrations exclude a mucinous compound.

The maspin concentrations are useful for grading and staging too. A tissular maspin concentration lower than 20 pg/mL can exclude a grade 3 tumor. For staging, if the tissular maspin concentration is lower than 40 pg/mL, the tumor is at stage 3/4, their distinction being given by the sum of tissular and saliva concentrations—if the sum is lower than 40 pg/mL, the tumor is at stage 3, elsewise the tumor at stage 4. A tissular maspin concentration higher than 40 pg/mL excludes a stage 4 tumor.

For TNM staging, pT = 4 in the next two circumstances:

- Whole-blood maspin concentrations lower than 55 pg/mL with tissular of saliva maspin concentrations higher than 40 pg/mL
- Whole-blood concentrations higher than 55 pg/mL with tissular maspin concentrations lower than 55 pg/mL and salivary maspin concentrations higher than 21 pg/mL.

Regarding the pN value, a whole-blood maspin concentration higher than 55 pg/mL can exclude pN = 3, while a whole-blood maspin concentration higher than 65 pg/mL with a urinary maspin concentration higher than 150 pg/mL or a salivary maspin concentration higher than 21 pg/mL can exclude pN = 2.

The final related features are invasions. For lymphatic invasion, the lymphatic maspin equation can be used to calculate Dist: if its value is lower than 1, there is no lymphatic invasion, while a Dist higher than 1 leads to a high probability of lymphatic invasion. Vascular invasion is related to whole-blood maspin concentration—a value lower than 50 pg/mL suggests the existence of an invasion, while a value higher than 50 pg/mL suggests the absence of an invasion. Perineural invasion is linked to whole-blood maspin concentration too: a maspin concentration value lower than 65 pg/mL suggests the existence of an invasion, while a maspin concentration value higher than 65 pg/mL suggests the absence of an invasion.

**Scheme 2:**
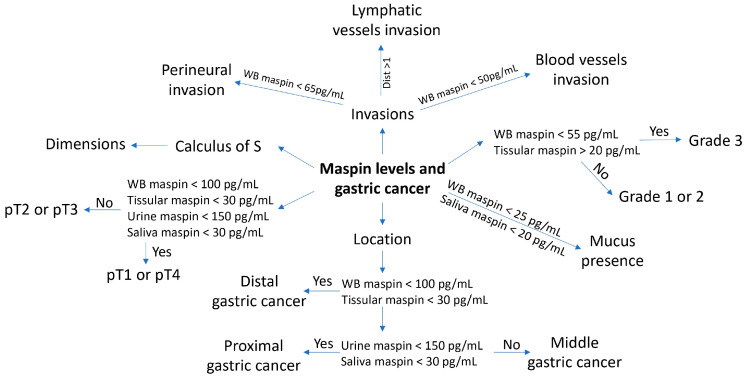
A simplified diagram of applications on clinicopathological features determination by using maspin levels in different biological features.

All of the abovepresented correlations can be easily observed in Scheme 2, which is given below.

As observed in Scheme 2, by using the levels of maspin in the four biological fluids (tissue, whole blood, saliva and urine), clinicopathological features can be anticipated with high probability.

## 5. Conclusions

Correlations between a series of pathological features and the maspin concentration in different biological samples have been found. These correlations may facilitate the minimally invasive and fast diagnosis of gastric cancer based on the reliable detection of maspin concentration in saliva, urine, and whole blood.

By using maspin levels in the four biological fluids, we developed algorithms for determination with a high probability of location (based on all four levels), TNM staging (also based on all four levels), size (based on whole-blood, tissue and urine levels), invasions (based on whole-blood levels), tumor grade (based on whole-blood and tissue levels) and mucus presence (based on whole-blood and saliva levels).

## Figures and Tables

**Table 1:** The number of patients counted as a function of the maspin concentration—for a proximal gastric cancer location.

Whole Blood		Tissue		Urine		Saliva	
Maspin (pg/mL)	Number of Patients	Maspin (pg/mL)	Number of Patients	Maspin (pg/mL)	Number of Patients	Maspin (pg/mL)	Number of Patients
<150	5	<150	8	<150	3	<150	8
>150	2	>150	0	>150	5	>150	0
<100	4	<100	6	<100	3	<100	8
>100	3	>100	2	>100	5	>100	0
>30	5	>30	5	>30	6	>30	2
<30	2	<30	3	<30	2	<30	6

**Table 2:** The number of patients counted as a function of the maspin concentration—for a middle gastric cancer location.

Whole Blood		Tissue		Urine		Saliva	
Maspin (pg/mL)	Number of Patients	Maspin (pg/mL)	Number of Patients	Maspin (pg/mL)	Number of Patients	Maspin (pg/mL)	Number of Patients
<150	6	<150	5	<150	4	<150	5
>150	1	>150	1	>150	2	>150	1
<100	4	<100	4	<100	4	<100	5
>100	3	>100	2	>100	2	>100	1
>30	6	>30	4	>30	5	>30	3
<30	1	<30	2	<30	2	<30	3

**Table 3:** The number of patients counted as a function of the maspin concentration—for a distal gastric cancer location.

Whole Blood		Tissue		Urine		Saliva	
Maspin (pg/mL)	Number of Patients	Maspin (pg/mL)	Number of Patients	Maspin (pg/mL)	Number of Patients	Maspin (pg/mL)	Number of Patients
<150	14	<150	14	<150	6	<150	11
>150	0	>150	2	>150	7	>150	1
<100	12	<100	14	<100	3	<100	11
>100	2	>100	2	>100	10	>100	1
>30	7	>30	3	>30	11	>30	3
<30	7	<30	13	<30	2	<30	9

**Table 4:** Classification of the cases studied according to the pT values and the concentration of maspin of 55 pg/mL taken as a reference concentration.

pT	Values (pg/mL)	Number of Patients			
		Whole Blood	Tumor Tissue	Urine	Saliva
1	<55	2	3	0	3
1	>55	1	0	4	1
2	<55	1	3	2	2
2	>55	2	1	1	2
3	<55	2	5	2	3
3	>55	5	3	4	2
4	<55	12	13	2	10
4	>55	1	2	10	2

## Data Availability

All data used for this study are available in the Supplementary Materials.

## Supplementary Materials

The following supporting information can be downloaded at: https://www.mdpi.com/article/10.3390/diagnostics13111857/s1, Figure S1. The correlation of maspin concentrations with the diameter of the tumor. Figure S2. Correlation of the whole-blood maspin concentrations with the pN values. Figure S3. Correlation of maspin whole-blood concentration with lymphatic invasion using: (a) values of maspin whole-blood concentrations; (b) calculated Dist parameter versus 180pg/mL whole-blood maspin as a reference whole-blood maspin concentration. Figure S4. Whole-blood maspin concentration correlated with vascular invasion. Figure S5. Correlation of the concentration of whole-blood maspin with perineural invasion. Table S1. Determination of maspin in different biological samples, using stochastic sensors and associated pathological features. Table S2. The average values for maspin concentrations determined in different biological samples versus the presence/absence of mucus. Table S3. Average values of whole-blood maspin concentrations correlated with the pN values.
