# Supplementary material for: Minimally Invasive and Fast Diagnosis of Gastric Cancer Based on Maspin Levels in Different Biological Samples
Source: Diagnostics (Basel). 2023 May 26;13(11):1857. doi: 10.3390/diagnostics13111857 (PMC10252817; doi:10.3390/diagnostics13111857)
Supplement: Supplementary file 1 [file diagnostics-13-01857-s001.zip › diagnostics-2377644-supplementary.pdf]

Supplementary Materials

Minimally Invasive and Fast Diagnosis of Gastric Cancer Based on Maspin Levels in Different Biological Samples

Alexandru Adrian Bratei [1,2] and Raluca-Ioana Stefan-van Staden [1,2,*]

[1] Faculty of Chemical Engineering and Biotechnologies, University Politehnica of Bucharest, 011061 Bucharest, Romania
[2] Laboratory of Electrochemistry and PATLAB, National Institute of Research for Electrochemistry and Condensed Matter, 060021 Bucharest, Romania
* Correspondence: ralucavanstaden@gmail.com

Table S1. Determination of maspin in different biological samples, using stochastic sensors, and associated pathological features.

Patient nr.	Whole blood	Tissue	Urine	Saliva	Location	Maximum diameter of tumor (mm)	Presence of mucinous compound	Grading	pT	pN	Stage	Lymphatic invasion	Vascular invasion	Perineural invasion
	Maspin (pg/mL)	Maspin (pg/mL)	Maspin (pg/mL)	Maspin (pg/mL)										
1	-	31.68±0.07	-	21.51±0.04	Proximal gastric tumor	-	Yes	-	4	2	4	Yes	Yes	Yes
2	375.73±0.03	105.82±0.01	1.41±0.02	69.06±0.03	Proximal gastric tumor	35	No	3	3	0	2	Yes	No	No
3	1.31±0.03	191.43±0.03	5.03±0.03	252.97±0.04	Distal gastric tumor	70	No	3	4	3	3	Yes	Yes	Yes
4	-	10.07±0.02	1843.4±0.03	23.74±0.03	Middle gastric tumor	165	No	-	4	3	3	Yes	Yes	Yes
5	35.6±0.03	30.2±0.05	1932.5±0.05	14.08±0.03	Distal gastric tumor	-	No	-	1	0	1	-	-	-
6	25.18±0.04	5.17±0.02	921.44±0.02	20.03±0.02	Proximal gastric tumor	24	No	3	4	3	3	Yes	Yes	Yes
7	116.51±0.02	180.64±0.01	9.41±0.01	55.13±0.02	Distal gastric tumor	35	No	2	4	1	3	Yes	Yes	Yes
8	100.88±0.05	21.33±0.04	445.56±0.02	-	Distal gastric tumor	40	-	-	3	0	2	No	No	Yes
9	39.34±0.02	-	-	-	Middle gastric tumor	147	-	-	4	3	3	Yes	Yes	Yes
10	-	46.03±0.05	275.51±0.03	22.03±0.04	Middle gastric tumor	28	No	3	4	0	2	Yes	Yes	No
11	293.9±0.03	42.31±0.04	8.83±0.05	56.43±0.03	Middle gastric tumor	25	-	-	2	0	1	Yes	No	No
12	60.95±0.03	31.61±0.04	92.64±0.04	-	Proximal gastric tumor	50	Yes	3	3	2	-	Yes	Yes	Yes
13	-	17.8±0.05	-	20.78±0.03	Distal gastric tumor	55	No	2	2	0	1	No	No	No

14	16.09±0.04	18.24±0.03	2834.88±0.04	22.25±0.03	Proximal gastric tumor	>100	No	-	4	3	4	Yes	Yes	Yes
15	-	11.26±0.05	-	-	Distal gastric tumor	60	Yes	-	4	2	4	Yes	Yes	Yes
16	165.5±0.03	8.8±0.03	-	-	Middle gastric tumor	15	No	2	3	0	-	No	No	No
17	16.95±0.04	-	1844.1±0.04	36.37±0.04	-	-	-	-	-	-	-	-	-	-
18	132.94±0.04	30.43±0.07	31.66±0.03	60.14±0.02	-	-	-	-	-	-	-	-	-	-
19	-	98.77±0.06	-	-	-	-	-	-	-	-	-	-	-	-
20	33.89±0.03	-	445.56±0.04	-	Proximal gastric tumor	9	-	-	-	-	-	-	-	-
21	22.63±0.02	60.88±0.05	283.38±0.03	8.02±0.04	Distal gastric tumor	45	Yes	3	3	0	2	Yes	Yes	Yes
22	123.41±0.02	482.79±0.02	5.45±0.03	252.97±0.03	Middle gastric tumor	20	No	-	2	0	1	Yes	No	Yes
23	37.41±0.03	24.3±0.03	-	22.48±0.04	Distal gastric tumor	65	No	-	4	3	4	Yes	No	Yes
24	44.54±0.05	-	-	-	Middle gastric tumor	-	Yes	-	4	3	3	Yes	No	No
25	5.17±0.04	39.34±0.04	99.54±0.03	6.65±0.01	Middle gastric tumor	-	-	-	4	3	-	-	-	-
26	22.85±0.03	16.46±0.06	336.47±0.02	11.85±0.02	Distal gastric tumor	-	No	2	2	0	-	No	Yes	No
27	52.52±0.04	21.14±0.06	-	-	Distal gastric tumor	100	No	3	4	3	-	Yes	No	Yes
28	19.06±0.02	38.6±0.05	149.36±0.05	7.4±0.02	Distal gastric tumor	50	No	3	4	2	-	Yes	No	Yes
29	46.27±0.05	20.77±0.04	137.67±0.04	13.96±0.05	Distal gastric tumor	-	-	3	4	3	-	-	-	-
30	28.57±0.05	12.72±0.04	641.56±0.03	22.25±0.03	Distal gastric tumor	24	No	-	4	1	-	Yes	Yes	Yes

31	18.24±0.02	18.29±0.05	94.81±0.04	98.65±0.03	Distal gastric tumor	50	Yes	-	1	0	-	Yes	No	Yes
32	24.3±0.03	16.61±0.07	101.55±0.03	11.11±0.04	Distal gastric tumor	100	Yes	2	3	0	-	No	Yes	No
33	228.55±0.03	130.67±0.03	2.66±0.03	57.53±0.03	Proximal gastric tumor	-	No	-	3	0	2	No	No	No
34	-	19.91±0.06	-	16.31±0.05	Proximal gastric tumor	55	Yes	2	3	3	3	Yes	No	No
35	33.23±0.04	-	57.12±0.04	37.85±0.04	Middle gastric tumor	-	-	-	-	-	-	-	-	-
36	-	21.51±0.04	336.1±0.04	-	Distal gastric tumor	45	No	2	4	3	3	Yes	No	Yes
37	46.77±0.05	-	280.27±0.03	-	Distal gastric tumor	50	No	2	4	3	4	Yes	Yes	Yes
38	117±0.02	47.51±0.03	562.16±0.05	13.08±0.04	Proximal gastric tumor	25	No	2	1	0	1	No	No	No
39	-	-	162.46±0.03	25.97±0.03	Proximal gastric tumor	15	No	2	1	0	1	No	No	No

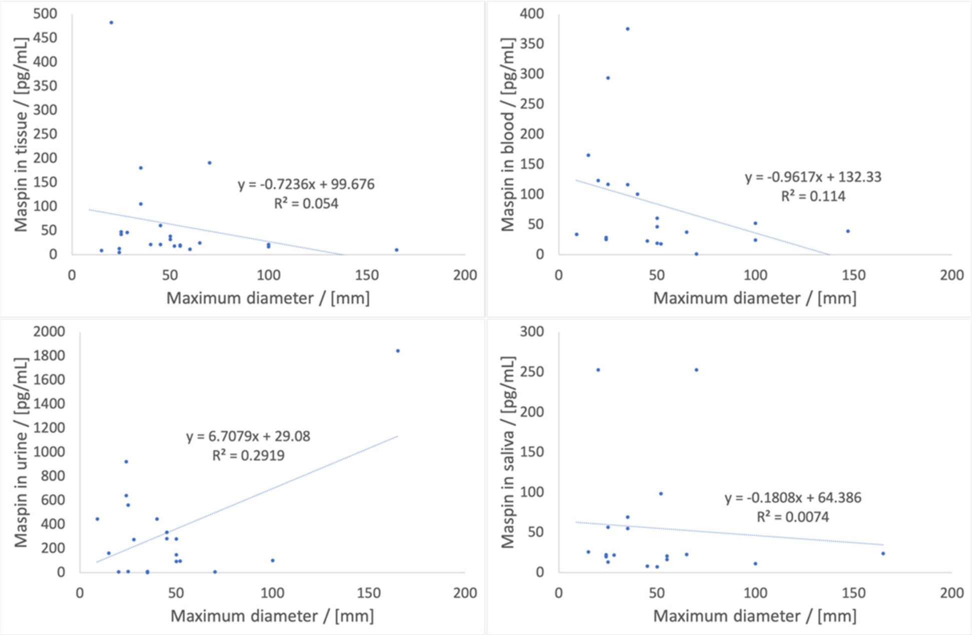

Figure S1. The correlation of maspin concentrations with the diameter of the tumor.

Table S2. The average values for maspin concentrations determined in different biological samples versus presence/absence of mucus.

Feature	Whole blood	Tissue	Urine	Saliva
No mucus	88.25	74.21	605.89	53.74
Present mucus	34.13	27.18	143.05	31.12

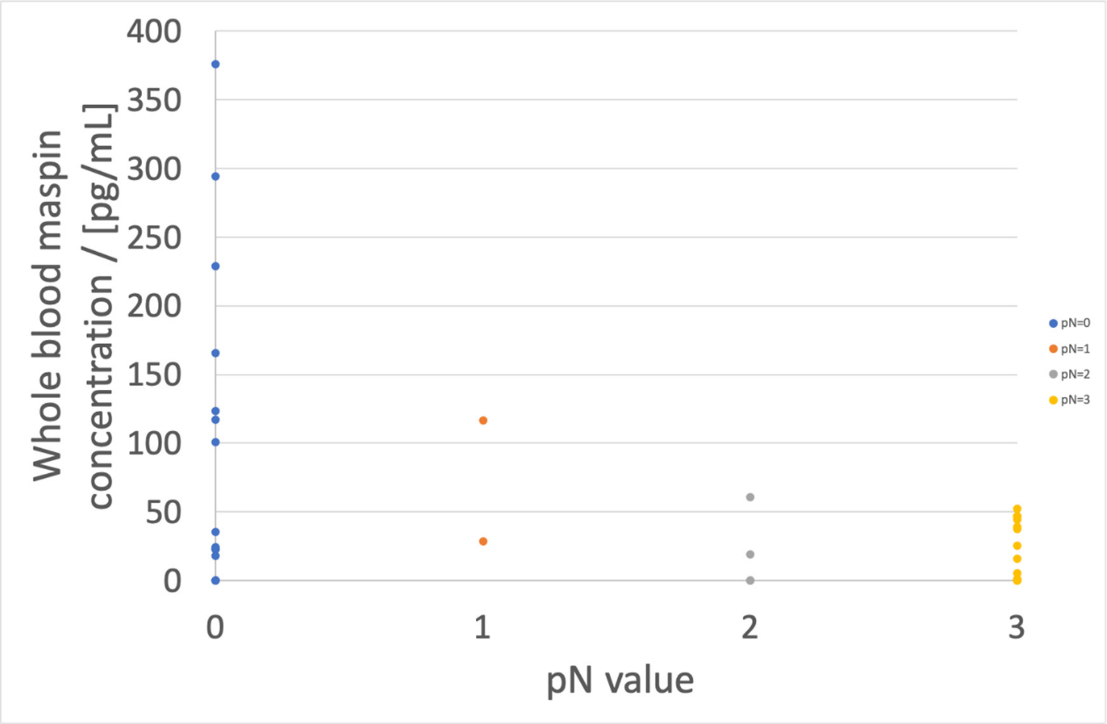

Figure S2. Correlation of the whole blood maspin concentrations with the pN values.

Table S3 Average values of whole blood maspin concentrations correlated with the pN values.

Whole blood maspin (pg/mL)	pN			
	0	1	2	3
	127.38	72.54	40.01	31.46

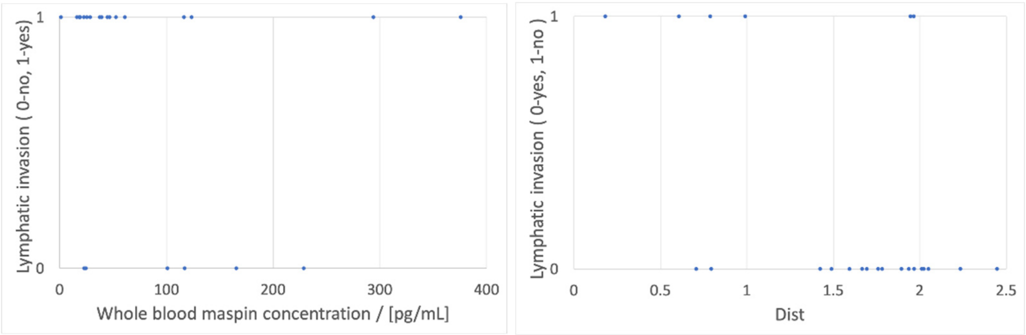

a **b**

Figure S3. Correlation of maspin whole blood concentration with lymphatic invasion using: a. values of maspin whole blood concentrations; b. Calculated Dist parameter versus 180pg/mL whole blood maspin as reference whole blood maspin concentration.

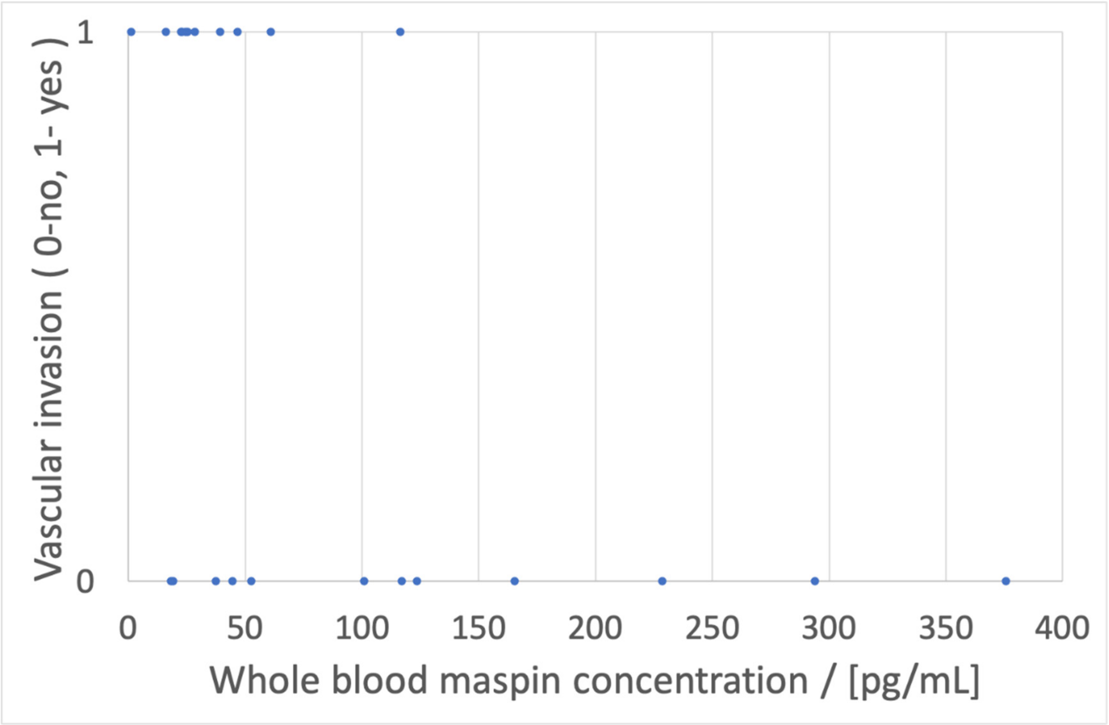

Figure S4. Whole blood maspin concentration correlated to vascular invasion.

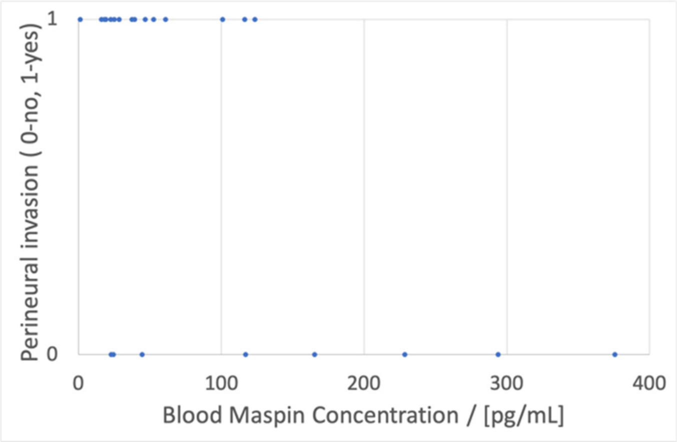

Figure S5. Correlation of the concentration of whole blood maspin with perineural invasion.
